# Targeting super-enhancers reprograms glioblastoma central carbon metabolism

**DOI:** 10.18632/oncotarget.27938

**Published:** 2021-06-22

**Authors:** Trang T.T. Nguyen, Mike-Andrew Westhoff, Georg Karpel-Massler, Markus D. Siegelin

**Affiliations:** ^1^Department of Pathology and Cell Biology, Columbia University Medical Center, New York, NY, USA; ^2^Department of Pediatrics and Adolescent Medicine, Ulm University Medical Center, Ulm, Germany; ^3^Department of Neurosurgery, Ulm University Medical Center, Ulm, Germany

**Keywords:** glioblastoma, metabolism, HDAC-inhibitor, c-Myc, fatty acid oxidation

## Abstract

The concept that tumor cells demand a distinct form of metabolism was appreciated almost a century ago when the German biochemist Otto Warburg realized that tumor cells heavily utilize glucose and produce lactic acid while relatively reducing oxidative metabolism. How this phenomenon is orchestrated and regulated is only partially understood and seems to involve certain transcription factors, including c-Myc, HIF1A and others. The epigenome eintails the posttranslational modification of histone proteins which in turn are involved in regulation of transcription. Recently, it was found that cis-regulatory elements appear to facilitate the Warburg effects since several genes encoding for glycolysis and associated pathways are surrounded by enhancer/super-enhancer regions. Disruption of these regions by FDA-approved HDAC inhibitors suppressed the transcription of these genes and elicited a reversal of the Warburg effect with activation of transcription factors facilitating oxidative energy metabolism with increases in transcription factors that are part of the PPARA family. Therefore, combined targeting of HDACs and oxidative metabolism suppressed tumor growth in patient-derived xenograft models of solid tumors, including glioblastoma.

## INTRODUCTION

Glioblastoma WHO IV (GBM) remains an incurable disease and an improved understanding of its metabolic properties may facilitate better treatments [1]. With the discovery of IDH1 mutant GBMs, deregulated metabolism became more relevant to study since IDH1 mutated GBMs substantially accumulate a certain metabolite, 2-HG, that in turn reprograms and shapes several aspects of cellular signaling [2, 3], especially related to global changes in the epigenome. However, there are many other reasons why metabolism is critical to study in GBMs, which for instance also includes the earlier observation that the core and the infiltrative margin of GBMs are distinct in their transcriptomic, proteomic and metabolic state [4]. In this regard, less prominent pathways, such as beta-oxidation of fatty acids, became more relevant in GBM biology. Moreover, stem-like GBM cells appear to have a reliance on both cholesterol and fatty acid synthesis [5, 6]. In this regard, we found that cholesterol regulates oxidative metabolism in GBM model systems [7, 8]. More recently, drug induced reprogramming of GBM metabolism have shown interesting findings, which includes a shift from glycolysis to oxidative metabolism following compound exposure. In this research perspective, we are briefly reviewing some basic and evolving aspects of GBM metabolism and highlight our recent findings on how the epigenome impacts the Warburg effect and oxidative energy metabolism in GBM.

### Aerobic glycolysis and its regulation in GBM by an enhancer/super-enhancer landscape

Almost a century ago, Otto Warburg appreciated the fact that tumor cells harbor a substantially reprogramed energy metabolism as compared to non-malignant cells. In this context, it was shown that glucose is rapidly metabolized to lactic acid, while under-utilizing the oxidative branch of metabolism, i.e., the TCA-cycle and the respiratory chain/oxidative phosphorylation. For these reasons, several transcripts regulating glycolysis are increased in tumor cells, including hexokinase 2 and LDHA [9]. Hexokinase 2 is critical in that it catalyzes the phosphorylation of glucose to glucose-6-phosphate, whereas LDHA catalyzes the final step in glycolysis from pyruvic acid to lactic acid [9]. These effects are regulated by several transcription factors, including c-Myc (*MYC)*, *HIF1A*, p53 (TP53) and others [9]. While only a relatively small fraction of GBMs displays MYC amplification, it’s overall levels are still increased in GBMs as compared to normal tissue based on the TCGA database. Moreover, stem-like GBM cells have significantly higher levels in c-Myc as compared to their differentiated counterparts [10]. Thus, there appears to be a role for c-Myc to impact GBM progression and resistance to therapy. In part, c-Myc is a facilitator of transcription of oncogenic genes by modulation of RNA-polymerase II phosphorylation via P-TEFb, which drives elongation of RNA-polymerase II [11]. Therefore, c-Myc is highly frequently localized to regions in the genome with open chromatin [11], which may involve cis-regulatory elements, i.e., enhancers and super-enhancer regions. Therefore, it becomes critical to dissect which down-stream effects are actually driver- and not passenger effects. The transcription factor c-Myc binds both to the promoter regions of hexokinase-2 and LHDA, respectively, supporting the notion that c-Myc is a driver of the Warburg effect in certain tumors [12]. In a classical experiment that uncovered the role of c-Myc as a driver for the Warburg effect it was demonstrated that c-Myc overexpression led to an enhanced production of lactic acid in fibroblasts, which was attributed and linked to the observation that c-Myc binds to the LDHA promoter to drive LDHA protein expression [12]. c-Myc is also involved in nucleotide-biosynthesis and regulates the expression of PHGDH, PSAT1 and PSPH, which are enzymes that connect glycolysis to serine/glycine biosynthesis and modulate one-carbon metabolism in tumors [11]. Although c-Myc is a regulator of these enzymes, there seems to be a complex interplay since other factors, e.g. the stress response transcription factor, ATF4, may also modulate their expression [13]. In this vein, our group made the recent observation that imipridones activate serine synthesis enzymes by up-regulation of ATF4, while at the same time imipridones suppress c-Myc protein levels in a manner reliant on GSK3β [13]. These observations exemplify the complexity of metabolism and many observations are context dependent. Moreover, both fatty acid and cholesterol synthesis are modulated by c-Myc expression, which was highlighted in stem-like GBM cells, recently [5, 6]. Finally, MYC appears to regulate histone acetylation and thereby affect the histone landscape [14]. Utilizing GBM stem-like cells, NCH644, we have performed chromatin immunoprecipitation with H3K27ac followed by next generation sequencing [15]. Employing a modified super-enhancer analysis (based on the previously published ROSE algorithm) we have found that GBM cells harbor super-enhancer and enhancer regions in enzymes and transporters involved in the Warburg-Effect, which included HK2, SLC2A1, ENO1, FASN and several others. In addition, a super-enhancer was found close to the MYC region, suggesting that high levels of c-Myc are driven by a super-enhancer in GBM stem like cells [15]. These epigenetic enriched regions (e.g., MYC and HK2) are preferentially found in GBM tumors over normal brain parenchyma [15].

### FDA-approved HDAC inhibitors affect the super-enhancer landscape of GBM cells

It was notable that these H3K27ac enriched regions displayed co-localization with HDAC2, suggesting that HDACs may be implicated in the anatomy of the enhancer and super-enhancer regions [15]. Therefore, we hypothesized that inhibition of HDAC2 by FDA-approved HDAC inhibitors, including romidepsin and panobinostat, may impact the formation of the super-enhancer landscape. Indeed, following treatment with both of these inhibitors the super-enhancer landscape was substantially changed in NCH644 stem-like GBM cells, which included disruption of super-enhancer regions related to MYC, HK2 and GAPDH, which resulted both in a suppression of mRNA and proteins related to these genes [15]. These observations are intriguing since it suggests that HDAC inhibitors might reduce tumor growth in part through blockage of the super-enhancer landscape. Our observations are consistent with results obtained by others in model systems of pediatric gliomas, although their findings did not focus on metabolism related genes and associated enhancers [16].

### HDAC inhibitors regulate the Warburg effect in part through modulation of the epigenome and through the transcription factor, c-Myc in model systems of GBM

We confirmed that these mRNA and protein changes indeed translated in metabolic changes by performing polar metabolite analysis. We found that the total levels of metabolites related to glycolysis as well as the pentose phosphate pathway were reduced, which suggested suppression of glycolysis and glucose feeding of the TCA-cycle [15]. Given that total levels of metabolites are not enough to conclude that a certain metabolic pathway is inhibited, we extended our studies further. We took a two prong strategy by extending our analysis to 13-C glucose tracing analyses as well as extracellular flux analysis. Glucose tracing analysis revealed that lactic acid was labeled by less glucose carbons following HDAC inhibitor exposure [15]. Similarly, extracellular acidification rate was reduced upon treatment with HDAC inhibitors in keeping with a reduction of glycolysis. Utilizing selective galactose culturing of GBM cells, we found evidence that HDAC inhibitors in part reduce glioma cell viability through inhibition of glycolysis, indicating that the observed interference with glycolysis is not merely a passenger effect [15]. Moreover, our glucose carbon tracing analysis pointed towards blockage of the pentose phosphate pathway and more globally interference with nucleotide biosynthesis, including flux from glucose to glycine [15]. We also noted a reduced labeling of TCA-cycle metabolites by glucose carbons even though our extracellular flux analysis revealed that while extracellular acidification rate is reduced, the oxygen consumption rate is increased following HDAC inhibitor treatment [15]. These findings are interesting because intuitively one would anticipate that when oxygen consumption rate is increased glucose oxidation is enhanced as well. However, our findings pointed towards a different fuel source to entertain this HDAC inhibitor mediated up-regulation of oxygen consumption. Given that c-Myc is a key regulator of glycolysis and our gene set enrichment analysis revealed substantial suppression of c-Myc targets as well as c-Myc by HDAC inhibitors in a manner dependent on HDAC1 and HDAC2 we linked HDAC inhibitor de-regulated carbohydrate metabolism and reduced viability with the associated decline of c-Myc transcript and protein levels. To this end, ectopic over-expression of c-Myc partially reversed viability loss and suppression of glycolysis induced by HDAC inhibitors [15].

### HDAC inhibitors elicit a pro-oxidative phenotype that in part is fueled by fatty acid oxidation

While suppressing glycolysis HDAC inhibitors drive oxidative phosphorylation in part through an increase of complexes of the respiratory chain [15]. Consequently, HDAC inhibitors and blockers of oxidative phosphorylation cause synergistic growth reduction in GBM model systems. In alignment with this observation, we found that the TRAP1 inhibitor, gamitrinib, enhanced the anti-glioma effects of HDAC inhibitors both *in vitro* and *in vivo*, which occurred in part through enhanced activation of a cell death with features of intrinsic apoptosis [17]. While our analyses did not demonstrate enhance glutamine oxidation, we pinpointed the fuel source for this phenomenon to increased fatty acid oxidation through utilization of 13-C uniformly labeled palmitic acid. These results need to be seen in the context with recent findings in GBM biology that propose a significant role of beta-oxidation in GBM [18–20]. For instance, recent data suggest a significant role of metabolic plasticity in the growth of GBM, enabling GBM cells to quickly switch from glycolysis to beta-oxidation [21]. Another fundamental principal is the notion that GBM harbor different regions/zones, which may be divided in the infiltrative margin or core [4]. While the core would be predicted to be more reliant on glycolysis, it is expected that the infiltrative margin should depend more on oxidative metabolism. This metabolic concept of core and infiltrative margin may be an important one since the ultimate progression of GBM will be determined by the migratory/infiltrative cells. By employing mass spectrometry imaging, recent work showed that the infiltrative margin of orthotopically injected GBM PDX cells in nude mice revealed elevated metabolites that are associated with fatty acid oxidation, suggesting that metabolization of lipids may be one of the key drivers of GBM progression and resistance to therapy [4]. These results should be viewed in concert with a recent study that showed that etomoxir extended animal survival in an orthotopic model system of GBM [22]. The limitation of the imaging study is that it only detected metabolites associated with fatty acid oxidation, but this would not ultimately prove enhanced oxidation of fatty acids. To this end, sophisticated *in vivo* tracer analyses would be necessary coupled with mass spectrometry imaging.

Referring to HDAC inhibitor reprogrammed GBM metabolism, we made the discovery that FDA-approved HDAC inhibitors in combination with etomoxir synergistically reduced the growth of a broad range of GBM model systems. The effect appeared to be less pronounced in astrocytes, suggesting a favorable toxicity profile [15]. Therefore, we extended these studies to *in vivo* model systems, involving GBM PDX model systems in the subcutaneous location, but more importantly in orthotopic models as well [15]. We found that the combination treatment of panobinostat and etomoxir extended animal survival more potently than single treatments, in keeping with the observation in cell culture [15]. Our studies are in line with similar studies in brain tumors as well as in other tumor entities that overall favor the usage/repurposing of etomoxir for oncological indications [22]. For instance, acute myeloid leukemia cells that were treated with standard of care chemotherapy reprogramed their metabolism to be more dependent on fatty acid oxidation and etomoxir counteracted this effect [23].

The oxidative metabolic reprogramming by HDAC inhibitors was partially orchestrated by at least two transcription factors that belong to the PPARA family. While we appreciated a suppression of c-Myc protein levels, a concomitant increase in PGC1A [24] and PPARD was noted. Both of these transcription factors were involved in mediating survival of GBM cells following HDAC inhibitor treatment. These effects are in keeping with earlier observations, showing that melanomas treated with BRAF inhibitors up-regulate PGC1A, which mediates resistance to these compounds [25]. Similarly, our own studies showed that GBM cells treated with c-MET inhibitor, crizotinib demonstrated an increase of PGC1A, resembling the findings observed with HDAC inhibitors and BRAF inhibitors [21]. Other have reported that PGC1A is down-stream of the mTOR signaling pathways in model systems of GBM. In this context, mTORC1 signaling was shown to facilitate oxidative metabolism which happened in part through modulation of PGC1A [26]. In addition to PGC1A, another member of the PPARA family, PPARD, was markedly induced upon HDAC inhibitor treatment. Unexpectedly, this transcription factor appeared to bear a substantial role in GBM survival since silencing of PPARD on its own affected GBM growth, suggesting that it may constitute a novel unexplored target on its own. Consistently, PPARD inhibition enhanced HDAC inhibitor mediated potency to reduce cellular viability.

In summary, while this perspective could not cover all emerging aspects of metabolism of GBMs, it is worthwhile noting that a better understanding of this process may likely lead to the design of more effective treatments for this still devastating disease. In this regard, improved patient stratification not related only to transcriptomic changes, but also encompassing metabolic alterations may be useful approaches for the future in this regard [27]. A more sophisticated understanding of carbon fuel requirements by GBMs may further increase our knowledge about these tumors. Finally, treatment induced metabolic changes also require more attention since drug treatments quickly change central carbon metabolism of tumor cells, supporting the use of metabolic drug combination therapies.

## Abbreviations

HIF1α: Hypoxia-inducible factor 1-alpha
PPARA: Peroxisome proliferator activated receptor alpha
HDAC: Histone deacetylase
IDH1: Isocitrate dehydrogenase 1
2-HG: α-Hydroxyglutaric acid
LDHA: Lactate dehydrogenase A
p53 (TP53): tumor suppressor p53
P-TEFb: The positive transcription elongation factor
PHGDH: Phosphoglycerate dehydrogenase
PSAT1: Phosphoserine aminotransferase 1
PSPH: Phosphoserine phosphatase
ATF4: Activating transcription factor 4
GSK3β: Glycogen synthase kinase 3 beta
SLC2A1: Solute carrier family 2, facilitated glucose transporter member 1
ENO1: Enolase 1
FASN: Fatty acid synthase
GAPDH: Glyceraldehyde-3-phosphate dehydrogenase
TRAP1: TNF receptor associated protein 1
PGC1A: Peroxisome proliferator-activated receptor gamma coactivator 1-alpha
PPARD: Peroxisome proliferator activated receptor delta
mTORC1: mammalian target of rapamycin complex 1
BRAF: proto-oncogene B-Raf

## References

[1] Neftel C , Laffy J , Filbin MG , Hara T , Shore ME , Rahme GJ , Richman AR , Silverbush D , Shaw ML , Hebert CM , Dewitt J , Gritsch S , Perez EM , et al. An Integrative Model of Cellular States, Plasticity, and Genetics for Glioblastoma. Cell. 2019; 178:835–849.e21. 10.1016/j.cell.2019.06.024. 31327527 PMC6703186

[2] Karpel-Massler G , Nguyen TTT , Shang E , Siegelin MD . Novel IDH1-Targeted Glioma Therapies. CNS Drugs. 2019; 33:1155–66. 10.1007/s40263-019-00684-6. 31768950 PMC7027940

[3] Karpel-Massler G , Ishida CT , Bianchetti E , Zhang Y , Shu C , Tsujiuchi T , Banu MA , Garcia F , Roth KA , Bruce JN , Canoll P , Siegelin MD . Induction of synthetic lethality in IDH1-mutated gliomas through inhibition of Bcl-xL. Nat Commun. 2017; 8:1067. 10.1038/s41467-017-00984-9. 29057925 PMC5651864

[4] Randall EC , Lopez BGC , Peng S , Regan MS , Abdelmoula WM , Basu SS , Santagata S , Yoon H , Haigis MC , Agar JN , Tran NL , Elmquist WF , White FM , et al. Localized Metabolomic Gradients in Patient-Derived Xenograft Models of Glioblastoma. Cancer Res. 2020; 80:1258–67. 10.1158/0008-5472.CAN-19-0638. 31767628 PMC7073296

[5] Gimple RC , Kidwell RL , Kim LJY , Sun T , Gromovsky AD , Wu Q , Wolf M , Lv D , Bhargava S , Jiang L , Prager BC , Wang X , Ye Q , et al. Glioma Stem Cell-Specific Superenhancer Promotes Polyunsaturated Fatty-Acid Synthesis to Support EGFR Signaling. Cancer Discov. 2019; 9:1248–67. 10.1158/2159-8290.CD-19-0061. 31201181 PMC6785242

[6] Wang X , Huang Z , Wu Q , Prager BC , Mack SC , Yang K , Kim LJY , Gimple RC , Shi Y , Lai S , Xie Q , Miller TE , Hubert CG , et al. MYC-Regulated Mevalonate Metabolism Maintains Brain Tumor-Initiating Cells. Cancer Res. 2017; 77:4947–60. 10.1158/0008-5472.CAN-17-0114. 28729418 PMC5600855

[7] Nguyen TTT , Ishida CT , Shang E , Shu C , Torrini C , Zhang Y , Bianchetti E , Sanchez-Quintero MJ , Kleiner G , Quinzii CM , Westhoff MA , Karpel-Massler G , Canoll P , et al. Activation of LXRβ inhibits tumor respiration and is synthetically lethal with Bcl-xL inhibition. EMBO Mol Med. 2019; 11:e10769. 10.15252/emmm.201910769. 31468706 PMC6783693

[8] Nguyen TTT , Ishida CT , Shang E , Shu C , Bianchetti E , Karpel-Massler G , Siegelin MD . Activation of LXR Receptors and Inhibition of TRAP1 Causes Synthetic Lethality in Solid Tumors. Cancers (Basel). 2019; 11:788. 10.3390/cancers11060788. 31181660 PMC6627953

[9] Mishra D , Banerjee D . Lactate Dehydrogenases as Metabolic Links between Tumor and Stroma in the Tumor Microenvironment. Cancers (Basel). 2019; 11:750. 10.3390/cancers11060750. 31146503 PMC6627402

[10] Wang J , Wang H , Li Z , Wu Q , Lathia JD , McLendon RE , Hjelmeland AB , Rich JN . c-Myc is required for maintenance of glioma cancer stem cells. PLoS One. 2008; 3:e3769. 10.1371/journal.pone.0003769. 19020659 PMC2582454

[11] Hsieh AL , Walton ZE , Altman BJ , Stine ZE , Dang CV . MYC and metabolism on the path to cancer. Semin Cell Dev Biol. 2015; 43:11–21. 10.1016/j.semcdb.2015.08.003. 26277543 PMC4818970

[12] Shim H , Dolde C , Lewis BC , Wu CS , Dang G , Jungmann RA , Dalla-Favera R , Dang CV . c-Myc transactivation of LDH-A: implications for tumor metabolism and growth. Proc Natl Acad Sci U S A. 1997; 94:6658–63. 10.1073/pnas.94.13.6658. 9192621 PMC21214

[13] Ishida CT , Zhang Y , Bianchetti E , Shu C , Nguyen TTT , Kleiner G , Sanchez-Quintero MJ , Quinzii CM , Westhoff MA , Karpel-Massler G , Prabhu VV , Allen JE , Siegelin MD . Metabolic Reprogramming by Dual AKT/ERK Inhibition through Imipridones Elicits Unique Vulnerabilities in Glioblastoma. Clin Cancer Res. 2018; 24:5392–406. 10.1158/1078-0432.CCR-18-1040. 30037819 PMC6214769

[14] Poole CJ , van Riggelen J . MYC-Master Regulator of the Cancer Epigenome and Transcriptome. Genes (Basel). 2017; 8:142. 10.3390/genes8050142. 28505071 PMC5448016

[15] Nguyen TTT , Zhang Y , Shang E , Shu C , Torrini C , Zhao J , Bianchetti E , Mela A , Humala N , Mahajan A , Harmanci AO , Lei Z , Maienschein-Cline M , et al. HDAC inhibitors elicit metabolic reprogramming by targeting super-enhancers in glioblastoma models. J Clin Invest. 2020; 130:3699–716. 10.1172/JCI129049. 32315286 PMC7324177

[16] Nagaraja S , Vitanza NA , Woo PJ , Taylor KR , Liu F , Zhang L , Li M , Meng W , Ponnuswami A , Sun W , Ma J , Hulleman E , Swigut T , et al. Transcriptional Dependencies in Diffuse Intrinsic Pontine Glioma. Cancer Cell. 2017; 31:635–652.e6. 10.1016/j.ccell.2017.03.011. 28434841 PMC5462626

[17] Nguyen TTT , Zhang Y , Shang E , Shu C , Quinzii CM , Westhoff MA , Karpel-Massler G , Siegelin MD . Inhibition of HDAC1/2 Along with TRAP1 Causes Synthetic Lethality in Glioblastoma Model Systems. Cells. 2020; 9:1661. 10.3390/cells9071661. 32664214 PMC7407106

[18] Kant S , Kesarwani P , Prabhu A , Graham SF , Buelow KL , Nakano I , Chinnaiyan P . Enhanced fatty acid oxidation provides glioblastoma cells metabolic plasticity to accommodate to its dynamic nutrient microenvironment. Cell Death Dis. 2020; 11:253. 10.1038/s41419-020-2449-5. 32312953 PMC7170895

[19] Cheng X , Geng F , Pan M , Wu X , Zhong Y , Wang C , Tian Z , Cheng C , Zhang R , Puduvalli V , Horbinski C , Mo X , Han X , et al. Targeting DGAT1 Ameliorates Glioblastoma by Increasing Fat Catabolism and Oxidative Stress. Cell Metab. 2020; 32:229–42.e8. 10.1016/j.cmet.2020.06.002. 32559414 PMC7415721

[20] Sperry J , Condro MC , Guo L , Braas D , Vanderveer-Harris N , Kim KKO , Pope WB , Divakaruni AS , Lai A , Christofk H , Castro MG , Lowenstein PR , Le Belle JE , et al. Glioblastoma Utilizes Fatty Acids and Ketone Bodies for Growth Allowing Progression during Ketogenic Diet Therapy. iScience. 2020; 23:101453. 10.1016/j.isci.2020.101453. 32861192 PMC7471621

[21] Zhang Y , Nguyen TTT , Shang E , Mela A , Humala N , Mahajan A , Zhao J , Shu C , Torrini C , Sanchez-Quintero MJ , Kleiner G , Bianchetti E , Westhoff MA , et al. MET Inhibition Elicits PGC1alpha-Dependent Metabolic Reprogramming in Glioblastoma. Cancer Res. 2020; 80:30–43. 10.1158/0008-5472.CAN-19-1389. 31694905 PMC6942623

[22] Lin H , Patel S , Affleck VS , Wilson I , Turnbull DM , Joshi AR , Maxwell R , Stoll EA . Fatty acid oxidation is required for the respiration and proliferation of malignant glioma cells. Neuro Oncol. 2017; 19:43–54. 10.1093/neuonc/now128. 27365097 PMC5193020

[23] Farge T , Saland E , de Toni F , Aroua N , Hosseini M , Perry R , Bosc C , Sugita M , Stuani L , Fraisse M , Scotland S , Larrue C , Boutzen H , et al. Chemotherapy-Resistant Human Acute Myeloid Leukemia Cells Are Not Enriched for Leukemic Stem Cells but Require Oxidative Metabolism. Cancer Discov. 2017; 7:716–35. 10.1158/2159-8290.CD-16-0441. 28416471 PMC5501738

[24] Frattini V , Pagnotta SM , Tala , Fan JJ , Russo MV , Lee SB , Garofano L , Zhang J , Shi P , Lewis G , Sanson H , Frederick V , Castano AM , et al. A metabolic function of FGFR3-TACC3 gene fusions in cancer. Nature. 2018; 553:222–27. 10.1038/nature25171. 29323298 PMC5771419

[25] Vazquez F , Lim JH , Chim H , Bhalla K , Girnun G , Pierce K , Clish CB , Granter SR , Widlund HR , Spiegelman BM , Puigserver P . PGC1α expression defines a subset of human melanoma tumors with increased mitochondrial capacity and resistance to oxidative stress. Cancer Cell. 2013; 23:287–301. 10.1016/j.ccr.2012.11.020. 23416000 PMC3708305

[26] Thiepold AL , Lorenz NI , Foltyn M , Engel AL , Divé I , Urban H , Heller S , Bruns I , Hofmann U , Dröse S , Harter PN , Mittelbronn M , Steinbach JP , Ronellenfitsch MW . Mammalian target of rapamycin complex 1 activation sensitizes human glioma cells to hypoxia-induced cell death. Brain. 2017; 140:2623–38. 10.1093/brain/awx196. 28969371

[27] Wang LB , Karpova A , Gritsenko MA , Kyle JE , Cao S , Li Y , Rykunov D , Colaprico A , Rothstein JH , Hong R , Stathias V , Cornwell M , Petralia F , et al, and Clinical Proteomic Tumor Analysis Consortium. Proteogenomic and metabolomic characterization of human glioblastoma. Cancer Cell. 2021 Feb 11:S1535-6108(21)00050-7. 10.1016/j.ccell.2021.01.006. [Epub ahead of print]. 33577785 PMC8044053

